# The American Joint Committee on Cancer staging (AJCC) Atlas, by Greene FL, Compton CC, Fritz AG, Shah JP, Winchester DP

**DOI:** 10.1186/1477-7800-3-34

**Published:** 2006-10-13

**Authors:** Gurpreet Singh-Ranger

**Affiliations:** 1 Department of General Surgery, Colchester General Hospital, Colchester, Essex, UK

The AJCC cancer staging atlas is the official publication of the American Joint Committee on Cancer, the world's foremost authority on cancer staging information.

This is the first edition of this book, created as a compendium to the AJCC Cancer Staging Manual and Handbook, now in their sixth editions.

**Figure 1:**
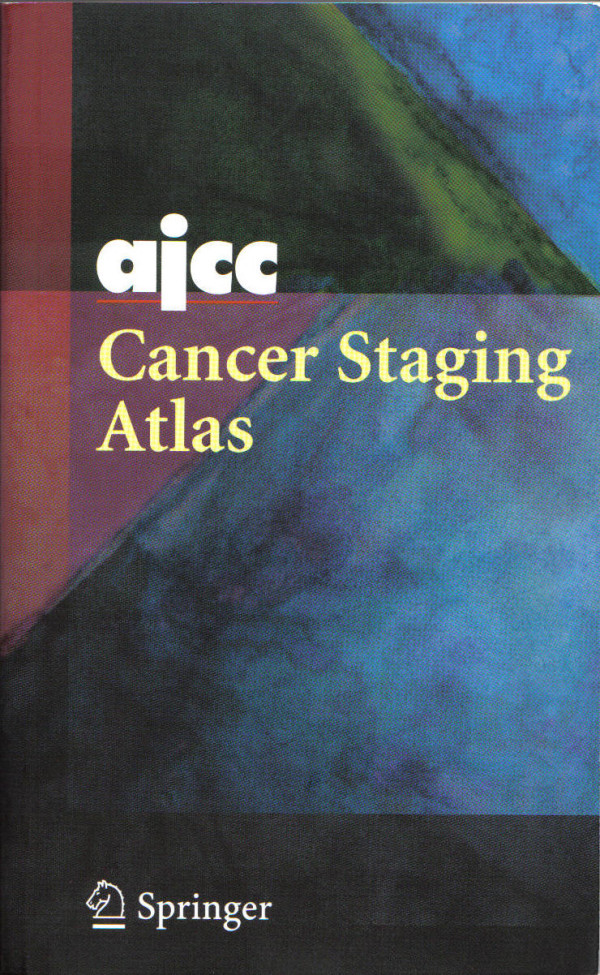
Front Cover of the Staging Atlas.

This is an impressive and extremely valuable small book, which is easily transportable due to its small size, 20 × 12.5 cm (Figure 1). It is also set out in a logical manner.

**Figure 2:**
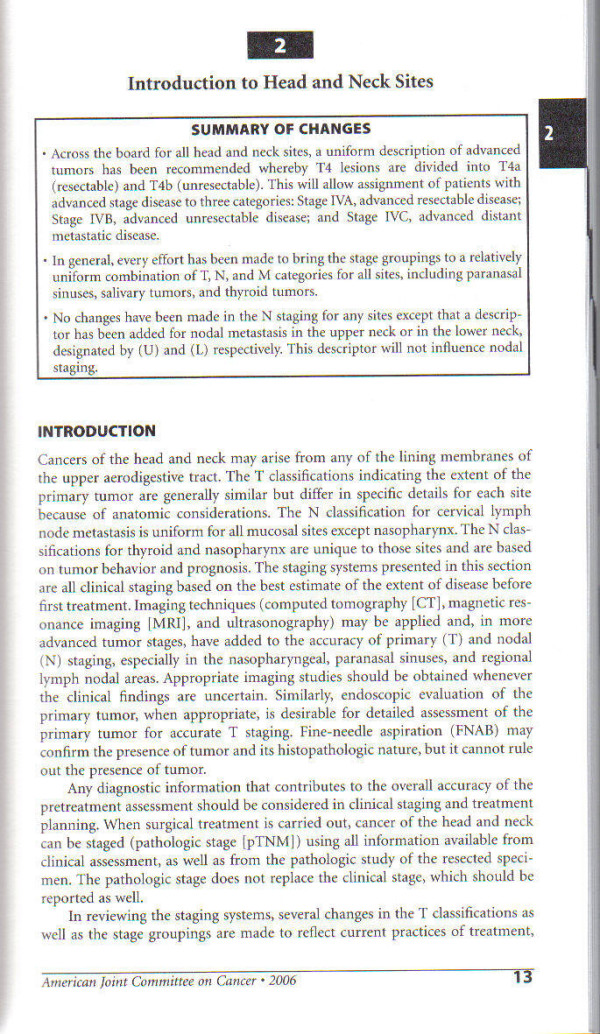
A Section Of The Atlas Detailing Layout And Ease Of Reference.

It contains over 400 illustrations to facilitate an understanding for the stage of a tumour, and can easily be referenced for individual patients. Each chapter is extremely detailed, and indexed at the page margin, so one can easily access the pertinent chapter in this way rather than going to the index or table of contents each time (Figure 2).

The atlas is divided into eight parts, covering all the major systems/sites of cancer, with an introductory chapter on the principles and purposes of staging. It is packed full of useful illustrations.

One of the most useful aspects of this atlas is the summary of changes for staging at the beginning of each system chapter (Figure 2).

I would recommend this publication to all health care professionals actively involved in the treatment of cancer patients, as the illustrations and text make the concept of the disease and its staging very simple to understand. It would also appeal to research students and post-doctoral scientists.

From a training perspective, the book is probably more suited to postgraduate oncology or surgery trainees.

